# A Retrospective Report of Carprofen Administration as Post-Operative Analgesia Reveals Negative Effects of Recommended Doses

**DOI:** 10.3390/ani14213157

**Published:** 2024-11-04

**Authors:** Zoë Jäckel, Ahmed Adžemović, Benedikt Kloos, Stefanie Hardung, Rita Sanchez-Brandelik, Philippe Coulon, Ilka Diester

**Affiliations:** 1 Optophysiology, Faculty of Biology, University of Freiburg, 79110 Freiburg, Germany; ahmed.adzemovic@biologie.uni-freiburg.de (A.A.); stefanie.hardung@biologie.uni-freiburg.de (S.H.); philippe.coulon@biologie.uni-freiburg.de (P.C.); 2 Intelligent Machine Brain Interfacing Technology (IMBIT)//BrainLinks-BrainTools, University of Freiburg, 79110 Freiburg, Germany; 3 Center for Experimental Models and Transgenic Service, University Medical Center Freiburg, 79104 Freiburg, Germany; 4 Faculty of Medicine, University of Freiburg, 79110 Freiburg, Germany; rita.sanchez@uniklinik-freiburg.de; 5 Bernstein Center Freiburg, University of Freiburg, 79104 Freiburg, Germany

**Keywords:** analgesia, laboratory animals, animal welfare, multimodal drug administration, stereotactic surgery, refinement

## Abstract

Effective pain management in laboratory animals is crucial for both animal welfare and the reliability of scientific research. We retrospectively examined the effects of carprofen as post-operative analgesia in Sprague Dawley rats following stereotactic surgery. Our data indicate that administering carprofen twice daily (5 mg/kg), as currently recommended by *Die Gesellschaft für Versuchstierkunde*/Society for Laboratory Animal Science (GV-SOLAS), led to adverse effects such as reduced food and water intake, disrupted fecal excretion, and abdominal bloating consistent with peritonitis. Continued administration exacerbated these symptoms, with post-mortem findings of intestinal obstructions and ulcers. However, when the frequency was reduced to once daily, such adverse symptoms were not observed. These results are based on incidental data collected from various neuroscientific experiments, resulting in small and uneven sample groups across various experimental cohorts. The inherent imbalances in these groups present challenges for statistical interpretation. While the findings suggest that less frequent carprofen use may reduce adverse effects, the surgical interventions and concurrent use of other drugs in these experiments likely exacerbated these outcomes. Further investigation into the interactions between carprofen, surgical stress, and other perioperative factors is needed to refine analgesia protocols in laboratory animals. Despite these limitations, these observations contribute to understanding analgesia protocols and may assist in improving animal welfare practices.

## Simple Summary

Ensuring proper pain relief in laboratory animals is vital for their welfare and for obtaining accurate scientific results. Our findings come from examining data collected during routine neuroscientific experiments on rats, where carprofen, a commonly used analgesic, was administered after brain surgery along with other drugs. We noticed that when carprofen was administered twice daily, some rats showed serious side effects, such as reduced eating and drinking, bloating, and signs similar to a severe condition called peritonitis. Further examination revealed blocked intestines and ulcers in these rats. However, when the dosage was reduced to once a day, these side effects did not occur. These observations are based on data from different experimental groups that were not initially designed to test carprofen’s safety. While these results were likely influenced by the combination of surgeries and other treatments, they suggest that less frequent use of carprofen may be safer. We hope these findings will help improve pain management in laboratory animals and encourage further research to refine care protocols.

## 1. Introduction

Proper pain management in laboratory species is a fundamental aspect of animal welfare. However, underdiagnosis and undertreatment of pain are considered institutional problems in experimental science [1,2]. While increasing analgesic doses or combining different drugs into a multimodal regimen can address this gap of unmitigated pain in animals, they can also introduce adverse effects that compromise animal well-being and physiological functions. For example, buprenorphine administration (0.05 mg/kg s.c.) has been associated with pica behavior in rats, particularly in Sprague Dawley rats [3,4]. Furthermore, the genetic differences among inbred laboratory rodent strains result in varied drug metabolism, posing additional challenges to drug dose refinement [5]. Thus, reports about the side effects of pain management agents are crucial to eliminating suboptimal procedures in experiment-specific regimens. In this context, we report on undesirable symptoms encountered after adapting a carprofen-based analgesia regimen to meet updated recommendations [6].

Carprofen is a nonsteroidal anti-inflammatory drug commonly used for postoperative analgesia in laboratory rats, yet limited pharmacokinetic data exist to support its dosing regimen [1]. Recent recommendations from the German Society for Laboratory Animal Science (GV-SOLAS) for carprofen use in rats suggest administering 2–5 mg/kg every 12–24 h to provide effective post-operative analgesia [6]. However, these guidelines are primarily based on efficacy studies, which may not adequately address the drug’s analgesic effectiveness and safety profile [1,7,8]. Such recommendations shape experimental protocol development in our facility, in collaboration with veterinarians and regulatory authorities. Factors influencing drug dose regimens for laboratory animals include increased stress levels induced by frequent interventions and practical considerations for experimenters. To accommodate these factors, our approved experimental procedure (in accordance with the German animal welfare act: TierSchG § 8, sub-section 1) allows for a range of 1–2 carprofen applications per day. While internal recommendations favored twice-daily analgesia to prevent periods of insufficient pain mitigation, adverse effects prompted veterinary consult and a shift to once daily dosing, as previously used in our laboratory.

This study aims to investigate the adverse effects observed with the current carprofen-based analgesia regimen, within the context of multimodal drug administration and surgical interventions in neuroscientific experiments. By examining these side effects, we seek to refine analgesia protocols to enhance animal welfare and reduce confounding variables that may affect experimental outcomes. Our findings underscore the need for further research into optimizing analgesic strategies to balance efficacy and safety for laboratory animals.

## 2. Materials and Methods

### 2.1. Ethical Statement

All results presented here are secondary outcomes of neuroscientific studies aimed at advancing our understanding of cognitive action control, with potential implications for improving movement disorder therapy. Rats are relevant translational models for such experiments due to cognitive homologies with humans, especially in relation to the prefrontal cortex [9,10]. At no point were medical problems deliberately induced through drug administration. All animal-related procedures were in accordance with directive 2010/63/EU of the European Parliament and of the Council on the protection of animals used for scientific purposes and were approved by the local authorities (Regierungs–präsidium Freiburg).

### 2.2. Animal Care and Monitoring

Wild-type Sprague Dawley^®^ rats (RjHan:SD, Janvier Labs, Le Genest-Saint-Isle, France) were group-housed in individually ventilated cages (480 × 375 × 210 mm, Eurostandard Type IV S, 1500U; TECNIPLAST, Hohenpeissenberg, Germany), with a maximum of 4 rats (up to 400 g), or 3 rats (up to 600 g) per cage. Cages were equipped with aspen wood bedding, unbleached egg cartons, paper towels, and aspen wood gnawing sticks for nesting and enrichment. Rats were maintained on a reversed 12 h light/dark cycle and had ad libitum access to food (#1314, 10 mm breeding diet pellets; Altromin International, Lage, Germany) and water, except for specific groups (Groups 3 and 4), which underwent behavioral training and were water-deprived during training sessions, with careful monitoring to maintain healthy body weight. No water deprivation occurred during the surgical or recovery periods. The rats were acclimatized to their new housing environment for a minimum of one day post-transport before any handling, and habituated to the environment for at least one week before surgical interventions. Animals were familiarized to experimenters via handling for at least three days before surgery to minimize stress during procedures, as approved by local authorities (see section below: Institutional Review Board Statement).

### 2.3. Stereotactic Surgery

All rats underwent stereotactic cranial surgery, and uniform drug administration was employed across all cohorts on the day of surgery (Table 1). Rats were briefly anesthetized with 5% isoflurane and received i.p. injections of 80 mg/kg ketamine and 0.06 mg/kg medetomidine. Prior to surgery, surgical analgesia was pre-emptively applied via subcutaneous injection of 0.05 mg/kg buprenorphine and local application of xylocaine gel to the incision site. A concluding s.c. injection of carprofen was administered immediately following the end of surgery as pre-emptive analgesia for post-surgical pain. Cohorts 1 and 2 underwent unilateral injection of an optogenetic virus (University of North Carolina Vector Core, Chapel Hill, NC, USA) and implantation with a low-profile optical fiber (200 µm-wide; Doric Lenses, Quebec, QC, Canada). Cohorts 3 and 4 underwent silicone electrode implantation (E32+R-100-S2-L6-200 NT, Atlas Neuroengineering, Leuven, Belgium). Cohort 5 received only viral injections without implantation. The latter were by far the shortest surgeries, did not involve complicated recoveries, and generally involved the least amount of stress to the animals (Table 2). For a more detailed description of the surgical techniques, please refer to previous studies from our group [11,12].

### 2.4. Post-Operative Care

**Figure 1:**
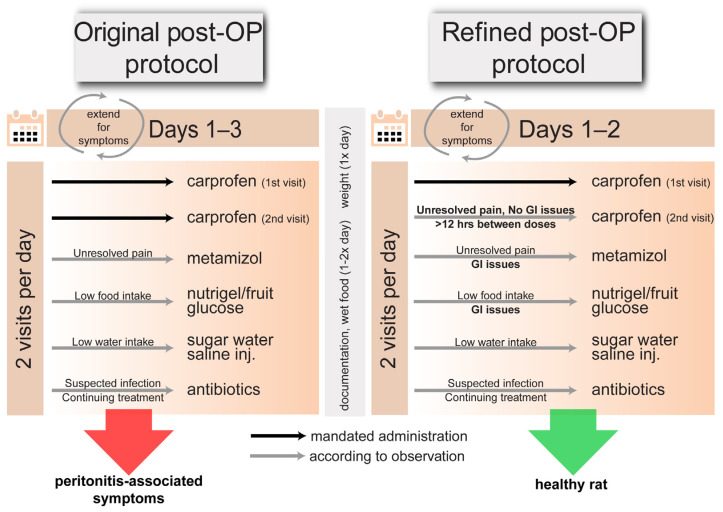
**Original analgesia protocol vs. refined analgesia protocol.** Schematic comparing the original analgesia protocol with the current refined model for our experimental purposes. The black arrows represent default carprofen administration per the approved timeline; the grey arrows indicate observation-based administration, including emergency treatments for unexpected symptom presentation (see post-operative care section). Both protocols include a minimum of a 3-day post-surgical observation, including a recording of the daily weight, as well as documentation of animal monitoring, including any symptoms of pain or discomfort, along with wet food supplementation; in both protocols, any possible extension of post-OP care is based on individual observations. The left column describes the original analgesia treatment, with carprofen automatically administered twice daily (spread across visits in the morning and evening) for 3 days post-OP. The right column displays the refined analgesia treatment, where carprofen is applied once daily for 2 days. Additional applications of carprofen can be further applied as needed, provided there is a minimum of 12 h between doses, and no gastrointestinal (GI) issues are found. In case of GI issues, carprofen is replaced with metamizole. The refined protocol tailors treatment to precise observations and considers carprofen’s impact on the gastric system. The original post-OP protocol resulted in high incidence of peritonitis-indicative symptoms, while refined post-OP protocols resulted in zero incidence of such symptom presentation.

To reduce post-surgical pain, 5 mg/kg of carprofen (Zoetis Deutschland GmbH, Berlin, Germany) was administered pre-emptively 1–2 times daily for 2–3 post-operative days (Table 1 and Table 2), following current recommendations [6]. For Groups A, C, and E, carprofen was prioritized per oral route (as this method is less stressful than injections) by hiding crushed tablets (25 mg Rimadyl^®^ chewable tablets, portioned to achieve a 5 mg/kg dose) in fruit or nutrigel (Nutriplus Gel; Virbac, Bad Oldesloe, Germany). The animals were monitored to ensure they consumed carprofen; when they did not, carprofen was instead administered by s.c. injection (see Supplementary Table S1). For the remaining groups, carprofen was consistently administered subcutaneously to reduce variability across subjects. To facilitate food intake, wet food was provided 1–2 times daily for up to 1.5 weeks post-surgery. The rats were observed twice daily for at least three days post-OP, and then once every three days for two weeks. The rats displaying abnormal or pain-related behavior were monitored daily until the symptoms subsided. To assess behavior and pain, the rats were remotely observed for general appearance (grooming condition, body posture), grimace scale, breathing rate, and spontaneous behavior. Further examination during handling and weighing included assessing locomotion, reaction to handling, interaction with the environment, hydration (via skin pinch), body condition, weight loss, and wound healing. The rat cages were examined to assess recovery based on nesting behavior and fecal excretion. In cases where animals exhibited negative post-operative symptoms, the faculty veterinarian conducted evaluations to verify the symptom assessment. The post-operative care window and the carprofen administration time period were extended for rats that displayed pain or discomfort, with particular attention to abnormal or severe symptoms. Suspected infections prompted a 20 mg/kg s.c. injection of an antibiotic (Sulphix; bela-pharm GmbH & Co. KG, Vechta, Germany) once daily (Figure 1). Antibiotic administration typically lasts for 3–5 days, but in these cases, the treatment ended with humane termination, resulting in a treatment duration of 1 to 5 days. Decreased food intake was addressed by motivating consumption with nutrigel, fruit, and sweetened water; if unsuccessful, 5% glucose (1 mL) was administered s.c. to maintain the blood sugar levels. For low water intake, saline (1–2 mL) was injected s.c. to prevent dehydration, while encouraging natural food and drink intake was prioritized over injections.

After observing several instances of negative outcomes, we suspected the high frequency carprofen administration to be exacerbating symptoms. We therefore modulated analgesia to lower-frequency carprofen administration, with ongoing documentation and veterinary consultation (within monitoring and diagnostic limitations) to optimize outcomes.

### 2.5. Protocol Modulation Between Rat Cohorts

The rats in this study belonged to separate cohorts, varying in age, sex, and the surgical procedures conducted for different neuroscientific experiments (Table 2). Here, we report on the unintended side effects stemming from similar protocols in our animal model. We compiled post-OP observational data from separate neuroscientific experiments to investigate common factors, potentially contributing to negative symptoms. All included observational data were collected over a 1-year timeframe from post-OP care following primary surgeries conducted on rats by experimenters with at least 6 months of experience in stereotactic surgery. Operations that did not meet these criteria and non-survival surgeries were excluded. Post-OP analgesia protocols (Table 1) were modified between surgeries due to the high incidence of adverse symptoms. As such, the treatments and post-OP observations were not randomized or blinded. To assess effects from the frequency of carprofen administration on rat health, rats were categorized post hoc into two groups: a high frequency carprofen group (Protocols A–D), which received carprofen twice daily for at least two days, and a low frequency carprofen group (Protocols E–F), which received doses once daily, except for one animal in protocol E, which received two doses on the first day only and was switched to single doses afterwards.

Cohort 1 was first treated with the high frequency carprofen protocol (Protocols A and C). After several cases of suspected peritonitis, possible risk factors were examined before commencing procedures on further animals. As elevated pica behavior was observed in several rats post-surgery, the consumption of frequently chewed, non-essential cage items were suggested risk factors for bezoar formation and ulceration through mechanical damage to the GI tract. Consequently, the cage environment was adjusted for the next cohorts (Cohorts 2–5) by removing enrichment items such as cardboard and paper towels during the post-surgery week and restricting access to plastic vents extending into the cage. As an additional precaution, i.p. injections administered for anesthesia prior to surgery were supervised by the faculty veterinarian to rule out incorrect injection techniques as a contributing factor to ulceration and peritonitis. Cohort 2 and Cohort 3 initially received high frequency carprofen analgesia (Protocols A–D). In response to additional cases of suspected peritonitis, administration frequency was reduced (Protocol E, F) for the remaining animals of Cohort 2 and 3, as well as subsequent cohorts 4 and 5, aiming to mitigate adverse symptoms suspected to be linked with high frequency carprofen application.

### 2.6. Termination and Post-Mortem Investigation

We established humane endpoints to minimize suffering in rats. Termination criteria included a ≥15% weight loss from the initial weight. The initial weight, adjusted over time for regained weight, ensured that the termination criteria account for the post-recovery phase and normal growth changes. Other criteria encompassed persisting symptoms, such as cramps, paralysis, abnormal breathing, irregular thermoregulation, vocalizing pain, reduced grooming, limited exploration, self-harm, and food refusal. Seven rats that did not recover after high frequency carprofen treatment were euthanized via 5% isoflurane anesthesia followed by an overdose of i.p. ketamine and xylazine (Rompun 2%; Elanco GmbH, Bad Homburg, Germany). Post-mortem investigation was performed on six of these rats; this involved ventral abdominal access using surgical scissors, the removal of the greater omentum, and the dissection of the abdominal organs. The seventh rat did not undergo necropsy, as the focus at that time was on post-operative care, and as we had already performed post-mortem examinations in six animals, we opted not to conduct additional procedures.

### 2.7. Analysis

We utilized custom MATLAB scripts to categorize rats into groups according to sex, carprofen protocol, and health outcome data. We investigated the effects of carprofen treatment and sex on the frequency of negative health outcomes (peritonitis-indicative symptoms) with Fisher’s exact test. Groups with significant effects were also checked for any differences in baseline weight (independent *t*-test), which could play a role in robustness to surgical intervention and drug therapy, potentially affecting health outcomes. We further examined the association between three different surgical interventions and symptomatic presentation using Fisher’s exact test [13].

## 3. Results

### 3.1. High Frequency Carprofen Effect on Peritonitis-like Symptoms

All rats in Cohort 1 received high frequency carprofen treatment. Twenty percent (2/10) exhibited symptoms such as weight loss, hunched posture, poor balance, reduced food and water intake, pica behavior, piloerection, and abdominal bloating. Following veterinary recommendations, carprofen and antibiotic administration was extended beyond the standard post-OP care timeframe for these two rats to manage pain symptoms, along with antibiotics to treat suspected infections underlying the observed disease progression. In both cases, the symptoms worsened, with additional symptoms such as low movement, a lack of feces in the cage, and increased abdominal bloating, prompting humane termination.

Although post-surgical pica behavior was restricted in the following cohorts, the peritonitis-like symptoms persisted. Fifty percent of the Cohort 2 rats (4/8) and 100% of the Cohort 3 rats (2/2) from the high frequency carprofen group exhibited similar symptoms (Table 2). Among these, five received extended carprofen treatment and supplementary antibiotics, leading to termination due to exacerbating symptoms. The last rat, upon showing signs of peritonitis, had its post-operative carprofen therapy discontinued and was administered metamizole as a substitute analgesic, without the use of antibiotics; this rat subsequently recovered (see Table 1, Protocol D). No adverse symptoms were observed in rats that were treated with low frequency carprofen (0/11). Peritonitis symptoms were observed in several animals across different cohorts that underwent surgery under identical aseptic conditions, with no evidence of temporal clustering. Symptomatic presentations occurred over an extended timeframe rather than in close succession, suggesting that the symptoms were not attributable to a singular technical issue, such as drug quality or contamination.

**Figure 2:**
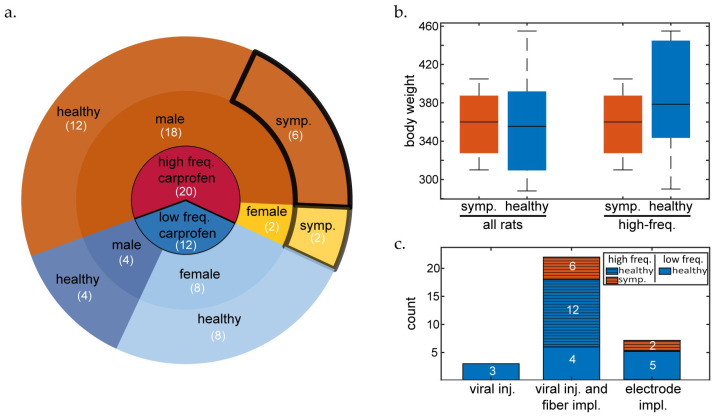
**Rat health status.** (**a**) Frequency of health status, treatment protocol, and sex. Blue shades: low frequency carprofen treatment; red/orange shades: high frequency carprofen treatment; brighter shades: female rats; medium shades: male rats; solid black outline: symptomatic (symp.) rats; no outline: healthy rats. The number of animals in each group is labelled in white text. (**b**) Box plot visualizing the distribution (median value: center bar; interquartile range: shaded box; minimum and maximum values: whiskers) of body weights of all symptomatic rats vs. all healthy rats (left) and symptomatic rats vs. healthy rats within the high frequency carprofen group. (**c**) Stacked bar plot visualizing the number of symptomatic and healthy animals per surgery type. The number of each surgery type and health outcome used to analyze the effect of surgery on health status is labelled in white on each bar. The bars are overlaid with a line pattern indicating where the animals underwent high frequency carprofen treatment.

In summary, 40% (8/20) of all high frequency carprofen-treated rats became symptomatic, with 35% (7/20) terminated due to peritonitis-like symptoms (initially observed 3–6 days post-surgery, after antibiotic administration and extended carprofen treatment commenced). Out of the female high frequency carprofen-treated rats, 100% (2/2) were affected by similar symptoms and were terminated. For males undergoing high frequency carprofen treatment, 33.33% (6/18) presented suspected peritonitis and 27.7% (5/18) were terminated due to the negative symptoms. The low frequency carprofen group rats presented zero events of suspected peritonitis (Figure 2).

We observed a significant effect of carprofen treatment (Figure 2a) on symptom presentation (Fisher’s exact test [two-tailed]: *p* = 0.0135). There was no significant effect between sex and health outcome (Fisher’s exact test [two-tailed]: *p* > 0.05). An independent *t*-test did not reveal significantly different initial body weights (Figure 2b) between symptomatic or healthy rats overall (mean difference: 1.8 g, *p* = 0.93, 95% CI: [−44.1889, 40.6056]), or within the high frequency carprofen group (mean difference: 28.5 g, *p* = 0.22, 95% CI: [−75.534, 18.6174]). In addition, we found no effect of surgery type (Figure 2c, Fisher’s exact test [two-tailed]: *p* = 0.84069) or carprofen administration route (Supplementary Table S2) on symptom presentation. As the sample sizes in this study were generally low, the groups assessed were particularly small and imbalanced, so we recommend caution when generalizing these findings.

### 3.2. Post-Mortem Analysis of High Frequency Carprofen-Treated Rats Reveals GI Complications

Peritonitis-like symptoms occurred in eight animals (all belonging to the high frequency carprofen protocols), six of which underwent post-mortem examination. Each rat presented signs of gastric defect and various signs of inflammatory or suppurative processes. Abdominal examination revealed abnormalities such as fluid-filled abdomen, discolored omental tissue, fecal obstructions up to 1.5 cm diameter in the large intestine, blood traces, intestinal discoloration, or intestinal ulceration.

## 4. Discussion

The 3Rs principles aims to replace animal experiments with alternative methods, reducing animal use, and refining procedures to minimize animal suffering. While adequate analgesia is crucial, its value diminishes if adverse effects from its administration lead to increased morbidity or suffering. Effective doses in animals, as in humans, may induce adverse effects [4], and careful monitoring for symptoms is essential, since animals cannot communicate discomfort directly. If drug protocols are not optimized, unnecessary suffering, increased animal usage, and repeated experiments may occur, thereby contradicting the 3Rs principles.

In our rat model, the high frequency carprofen treatment led to a substantial increase in peritonitis-like symptoms, with an unacceptably high (40%) percentage of animals showing signs of adverse effects. Antibiotics did not ameliorate the symptoms, consistent with previous reports of negative interactions resulting from the co-administration of carprofen and antibiotics [14,15]. In one case, metamizole successfully provided alternative analgesia while allowing gastric issues to subside.

The underlying mechanisms of frequent carprofen-induced gastric dysfunction in our study remain unclear. Due to the original study design, which was not intended as an analgesia analysis, and legislative regulations, we did not perform blood analyses, making it impossible to measure carprofen plasma levels to confirm sufficient analgesia, or plasma components such as cytokines to assess whether the observed peritonitis-consistent symptoms were linked to an excessive immune response. While blinding was not feasible due to the experimental design, objective criteria for symptom assessment were applied to minimize potential observer bias. As the subjects underwent variable interventions, drawing specific conclusions regarding interactions among sex, age, weight, or surgical intervention in relation to negative symptoms does not seem feasible. However, the post-hoc segregation of analgesia protocols was based on objective dosing records and indicates a trend for negative symptoms associated with twice-daily carprofen administration.

Our results suggest that a high frequency carprofen regimen (5 mg/kg, twice daily) within a multimodal anesthesia and analgesia protocol (involving isoflurane, ketamine, medetomidine, buprenorphine, lidocaine, and carprofen) may predispose animals to abnormal gastric function and higher morbidity rates. In contrast, reducing the carprofen administration frequency under the same multimodal drug protocol for cranial surgery resulted in a 100% recovery rate and healthy post-OP presentation, suggesting that less frequent carprofen is safer in our experimental context. These findings are supported by studies showing that carprofen interferes with anastomotic wound repair in the rat ileum [14], and that buprenorphine exacerbates ethanol-induced gastric lesions [15]. Considering their individual adverse effects on the gastric system, combination therapy including both carprofen and buprenorphine could elevate the risk of peritonitis. Indeed, a recent study revealed heightened drug sensitivity under multimodal therapy [16]. Multimodal drug-therapy (0.1 mg/kg of buprenorphine every 8 h, 5 mg/kg of carprofen every 24 h, and local irrigation during surgery with 10 mg/mL lidocaine and 2.5 mg/mL bupivacaine) led to peritonitis, while single-drug therapy with the same dose-frequency administration of carprofen or buprenorphine did not reflect such effects, nor did multimodal therapy with lower-dose buprenorphine [16]. Another recent study failed to find the desired synergies from multimodal drug application, potentially due to high carprofen doses (well-suited for monotherapy). They suggest that potential synergies of drug combinations in perioperative management should be assessed with lower carprofen doses [16]. Thus, current recommendations for high frequency administration may be suboptimal under specific strain-, experiment-, and multimodal-specific conditions.

These findings underscore the necessity of understanding the unintended side effects associated with these multimodal regimens, particularly in relation to surgical interventions that introduce additional stressors. While our report does not provide a comprehensive study, it contributes valuable insights into the complexities of pain management in animal research. Additionally, we argue for alternative analgesic strategies, particularly in the presence of gastric issues, to prevent symptom exacerbation. Effective analgesia and increased awareness of potential adverse effects from multimodal drug administration are crucial for optimizing animal care and aligning with the 3Rs principles.

## 5. Conclusions

Our findings suggest that high frequency carprofen administration in multimodal anesthesia and analgesia protocols may lead to significant adverse effects in rats, such as peritonitis-like symptoms and increased mortality. Reducing the frequency of carprofen administration resulted in better outcomes, emphasizing the need for careful consideration of drug protocols in animal models to prevent unnecessary suffering. Future research should further investigate the underlying mechanisms of drug interactions in multimodal settings and explore alternative analgesic options to enhance animal welfare and adhere to the 3Rs principles.

## Figures and Tables

**Table 1:** **Multimodal drug protocols.** Drug administration on the surgery day was identical across all cohorts. The rats were briefly anesthetized with 5% isoflurane (iso) and then administered i.p. injections of 80 mg/kg ketamine (ket) and 0.06 mg/kg medetomidine (med). An s.c. injection of 0.05 mg/kg buprenorphine (bup) was applied before surgery. Xylocaine gel (xyl) was applied to the skin above the skull incision site. Anesthesia was maintained during surgery at 0–3% isoflurane and 0.5 l/min O_2_. One s.c. carprofen (car) injection was administered at the end of surgery. Each carprofen dose was 5 mg/kg. Protocols A, C, and E substituted s.c. with per oral (p.o.) administration when possible (see Supplementary Materials). Protocols B, D, and F only included carprofen via s.c. injection. In some cases, observation-based extension of post-operative (post-OP) care was applied for as many days (X) deemed necessary past the general 3-day regimen. Protocol D substituted carprofen with 200 mg/kg metamizole (mm) administered orally directly to the mouth with a syringe during the extended care period. Protocols A–D were categorized as high frequency carprofen treatment (shaded in red), with twice daily carprofen for at least two out of three days post-OP. Protocols E and F were categorized as low frequency carprofen treatment (shaded in blue), with carprofen application once per day for up to three days post-OP, except for one animal in protocol E, which received a twice-daily dose only on the first day post-OP, but was switched to a single dose afterwards, --: no administration.

Group	Protocol	Day of Surgery		Post-OP Day 1		Post-OP Day 2		Post-OP Days 3–X	
		Pre-Surgery	Post-OP	Morn	Eve	Morn	Eve	Morn	Eve
**High frequency**	**A**	bup, ket, med, xyl, iso	car s.c.	car (p.o. or s.c.)	car (p.o. or s.c.)	car (p.o. or s.c.)	car (p.o. or s.c.)	car (p.o. or s.c.)	car (p.o. or s.c.)
	**B**	bup, ket, med, xyl, iso	car s.c.	car s.c.	car s.c.	car s.c.	car s.c.	car s.c.	car s.c.
	**C**	bup, ket, med, xyl, iso	car s.c.	car s.c.	car s.c.	car s.c.	car s.c.	car p.o.	--
	**D**	bup, ket, med, xyl, iso	car s.c.	car s.c.	car s.c.	car s.c.	car s.c.	mm p.o.	mm p.o.
**Low frequency**	**E**	bup, ket, med, xyl, iso	car s.c.	car p.o.	car p.o.	car p.o.	--	car p.o.	--
	**F**	bup, ket, med, xyl, iso	car s.c.	car s.c.	--	car s.c.	--	car s.c.	--

**Table 2:** **Categorization of animal cohorts.** Rats are separated by experimental cohort, defined by sex (male, female), age in weeks (w), body weight in grams (g) at surgery, and surgical procedure, and categorized as high- or low frequency carprofen-treated rats according to the post-OP protocol (Table 1). The rats from each cohort are categorized by the given post-OP protocols (Table 1) into high frequency carprofen (shaded in red) or low frequency carprofen groups (shaded in blue), along with the frequency of suspected peritonitis and early termination due to symptom severity. --: no administration.

Cohort	Sex	Age (w)	Body Weight (g)	N	Surgical Procedure	Protocol: N	Suspected Peritonitis	Early Termination
**1**	Male	8–9	375–475	10	Viral injection + low-profile fiber implantation	A: n = 9	2	2
						C: n = 1	0	0
**2**	Male	7–8	290–405	12	Viral injection + low-profile fiber implantation	A: n = 3	0	0
						B: n = 4	3	3
						D: n = 1	1	0
						F: n = 4	0	0
**3**	Female	>30	320–380	3	Electrode implantation	A: n = 2	2	2
						E: n = 1	0	0
**4**	Female	18	297–340	4	Electrode implantation	F: n = 4	0	0
**5**	Female	8–10	290–300	3	Viral injection	F: n = 3	0	0

## Data Availability

Detailed experimental methods, post-OP, and post-mortem documentation, as well as the analyses, are available upon reasonable request.

## Supplementary Materials

The following supporting information can be downloaded at: https://www.mdpi.com/article/10.3390/ani14213157/s1, Table S1: Group A carprofen administration protocol; Table S2: Health outcome vs. carprofen administration route.
