# Supplementary material for: A Retrospective Report of Carprofen Administration as Post-Operative Analgesia Reveals Negative Effects of Recommended Doses
Source: Animals (Basel). 2024 Nov 4;14(21):3157. doi: 10.3390/ani14213157 (PMC11544877; doi:10.3390/ani14213157)
Supplement: Supplementary file 1 [file animals-14-03157-s001.zip › animals-3236958-supplementary.pdf]

Supplementary Table S1: Group A carprofen administration protocol. Detailed documentation of carprofen administration routes for each animal in Group A, in Cohorts 1 and 2 during the first three post-operative days. Symptoms of peritonitis were observed (for rats 69 and 70, indicated with red text) within this timeframe, making it the most relevant period for evaluating the impact of analgesic treatment. Group A consisted of 12 animals, primarily receiving oral doses of carprofen via chewable tablets hidden in food to minimize stress from injections. In instances where an animal refused to eat the tablet, a subcutaneous (s.c.) injection was administered.

Cohort	Rat #	Day 1 morn	Day 1 eve	Day 2 morn	Day 2 eve	Day 3 morn	Day 3 eve
1	69	s.c.	s.c.	s.c.	s.c.	s.c.	p.o.
	70	s.c.	s.c.	p.o.	p.o.	p.o.	p.o.
	66	s.c.	s.c.	s.c.	p.o.	s.c.	p.o.
	65	s.c.	p.o.	p.o.	p.o.	p.o.	p.o.
	64	s.c.	s.c.	s.c.	s.c.	p.o.	p.o.
	67	p.o.	p.o.	p.o.	p.o.	p.o.	p.o.
	68	s.c.	s.c.	p.o.	p.o.	p.o.	p.o.
	62	s.c.	s.c.	s.c.	p.o.	p.o.	p.o.
	63	s.c.	s.c.	s.c.	p.o.	p.o.	p.o.
2	80	s.c.	s.c.	s.c.	s.c.	s.c.	p.o.
	71	s.c.	s.c.	s.c.	s.c.	s.c.	p.o.
	74	s.c.	s.c.	s.c.	s.c.	s.c.	p.o.

Supplementary Table S2: Health outcome vs. carprofen administration route. Amount of high carprofen frequency-treated animals receiving a mix of per oral and subcutaneous carprofen (Group A; see also Supplementary Table 1) vs. subcutaneous-only administration (Group B; see also Table 1). As both groups received carprofen twice daily for at least three post-operative days (during which the animals presenting peritonitis-consistent symptoms were identified), we compared the amount of symptomatic animals in each group for indications if administration route was related to negative post-OP state. No conclusive pattern or trend (Fisher's exact test [two-tailed]: p = 0.2451, Confidence Interval: [0.0105 1.6956]) for post-operative outcome in relation to carprofen administration route in the post-operative window was identified. However, it should be noted that the sample groups are small and uneven, and this should not rule out that administration route may be related to post-operative animal state. Here, no animals in the low-frequency carprofen group presented peritonitis-consistent symptoms, regardless of administration route (see also Table 2), suggesting that administration route is not a deciding factor for post-operative outcome.

High Carprofen frequency	Mixed (p.o. and s.c.) administration route	s.c. only
**peritonitis-consistent symptoms**	4	3
**healthy**	10	1
